# The Clinical Frailty Scale for mortality prediction of old acutely admitted intensive care patients: a meta-analysis of individual patient-level data

**DOI:** 10.1186/s13613-023-01132-x

**Published:** 2023-05-03

**Authors:** Raphael Romano Bruno, Bernhard Wernly, Sean M. Bagshaw, Mark van den Boogaard, Jai N. Darvall, Lina De Geer, Pablo Ruiz de Gopegui Miguelena, Daren K. Heyland, David Hewitt, Aluko A. Hope, Emilie Langlais, Pascale Le Maguet, Carmel L. Montgomery, Dimitrios Papageorgiou, Philippe Seguin, Wytske W. Geense, J. Alberto Silva-Obregón, Georg Wolff, Amin Polzin, Lisa Dannenberg, Malte Kelm, Hans Flaatten, Michael Beil, Marcus Franz, Sigal Sviri, Susannah Leaver, Bertrand Guidet, Ariane Boumendil, Christian Jung

**Affiliations:** 1 Medical Faculty, Department of Cardiology, Pulmonology and Vascular Medicine, Heinrich-Heine-University Duesseldorf, Moorenstraße 5, 40225 Duesseldorf, Germany; 2 Department of Internal Medicine, General Hospital Oberndorf, Teaching Hospital of the Paracelsus Medical Private University, Paracelsusstraße 37, 5110 Oberndorf, Austria; 3 Institute of General Practice, Family Medicine and Preventive Medicine, Paracelsus Medical University, Strubergasse 21, 5020 Salzburg, Austria; 4 Department of Critical Care Medicine, Faculty of Medicine and Dentistry, University of Alberta, and Alberta Health Services, 2-124 Clinical Sciences Building, 8440 112Th ST, Edmonton, AB T6G 2B7 Canada; 5 Department of Intensive Care Medicine, Radboud Institute for Health Sciences, Radboud University Medical Center, Nijmegen, The Netherlands; 6 Intensive Care Unit and Department of Anaesthesia & Pain Management, The Royal Melbourne Hospital, Grattan Street, Parkville, VIC 3050 Australia; 7 Department of Anaesthesiology and Intensive Care, Linköping University Hospital, Linköping, Sweden; 8 Hospital Universitario Miguel Servet, Saragossa, Spain; 9 Clinical Evaluation Research Unit, and Department of Critical Care Medicine, Queen’s University, Kingston, ON Canada; 10 Glasgow Royal Infirmary Intensive Care Unit, Glasgow, Scotland; 11 Division of Pulmonary, Allergy, and Critical Care Medicine, Oregon Health and Science University, Portland, OR USA; 12 Réanimation Chirurgicale, CHU Rennes, Université Rennes 1, Rennes, France; 13 Département d‘Anesthésie Réanimation, CHU Rennes, Rennes, France; 14 Service d’Anesthésie, CH Quimper, Quimper, France; 15 Faculty of Nursing, University of Alberta, Edmonton Clinic Health Academy, 3-171, Edmonton, AB T6G 1C9 Canada; 16 Faculty of Health and Caring Sciences Department of Nursing, University of West Attica (UWA) Athens, Egaleo, Greece; 17 Department of Intensive Care Medicine, Radboud Institute for Health Sciences, Radboud University Medical Center, Nijmegen, The Netherlands; 18 Department of Intensive Care Medicine, Hospital Universitario de Guadalajara, Guadalajara, Spain; 19 CARID (Cardiovascular Research Institute Düsseldorf), University Hospital of Düsseldorf, Germany, Düsseldorf, Germany; 20 Department of Clinical Medicine, Department of Anaesthesia and Intensive Care, University of Bergen, Haukeland University Hospital, Bergen, Norway; 21 Dept. of Medical Intensive Care, Hadassah Medical Center and Faculty of Medicine, Hebrew University of Jerusalem, Jerusalem, Israel; 22 Clinic of Internal Medicine I, Department of Cardiology, Friedrich Schiller University, 07737 Jena, Germany; 23 Department of Medical Intensive Care, Hadassah Medical Center and Faculty of Medicine, Hebrew University of Jerusalem, Jerusalem, Israel; 24 General Intensive Care, St George’s University Hospitals NHS Foundation Trust, London, UK; 25 Equipe: Épidémiologie Hospitalière Qualité Et Organisation Des Soins, Sorbonne Universités, UPMC Univ Paris 06, INSERM, UMR_S 1136, Institut Pierre Louis d’Epidémiologie Et de Santé Publique, 75012 Paris, France; 26 Service de Réanimation Médicale, Hôpitaux de Paris, Hôpital Saint-Antoine, 75012 Paris, France

**Keywords:** Frailty, Intensive care medicine, Outcome prediction, Systematic review, Individual patient-level data meta-analysis, Elderly

## Abstract

**Background:**

This large-scale analysis pools individual data about the Clinical Frailty Scale (CFS) to predict outcome in the intensive care unit (ICU).

**Methods:**

A systematic search identified all clinical trials that used the CFS in the ICU (PubMed searched until 24th June 2020). All patients who were electively admitted were excluded. The primary outcome was ICU mortality. Regression models were estimated on the complete data set, and for missing data, multiple imputations were utilised. Cox models were adjusted for age, sex, and illness acuity score (SOFA, SAPS II or APACHE II).

**Results:**

12 studies from 30 countries with anonymised individualised patient data were included (n = 23,989 patients). In the univariate analysis for all patients, being frail (CFS ≥ 5) was associated with an increased risk of ICU mortality, but not after adjustment. In older patients (≥ 65 years) there was an independent association with ICU mortality both in the complete case analysis (HR 1.34 (95% CI 1.25–1.44), p < 0.0001) and in the multiple imputation analysis (HR 1.35 (95% CI 1.26–1.45), p < 0.0001, adjusted for SOFA). In older patients, being vulnerable (CFS 4) alone did not significantly differ from being frail. After adjustment, a CFS of 4–5, 6, and ≥ 7 was associated with a significantly worse outcome compared to CFS of 1–3.

**Conclusions:**

Being frail is associated with a significantly increased risk for ICU mortality in older patients, while being vulnerable alone did not significantly differ. New Frailty categories might reflect its “continuum” better and predict ICU outcome more accurately.

*Trial registration:* Open Science Framework (OSF: https://osf.io/8buwk/).

**Graphical Abstract:**

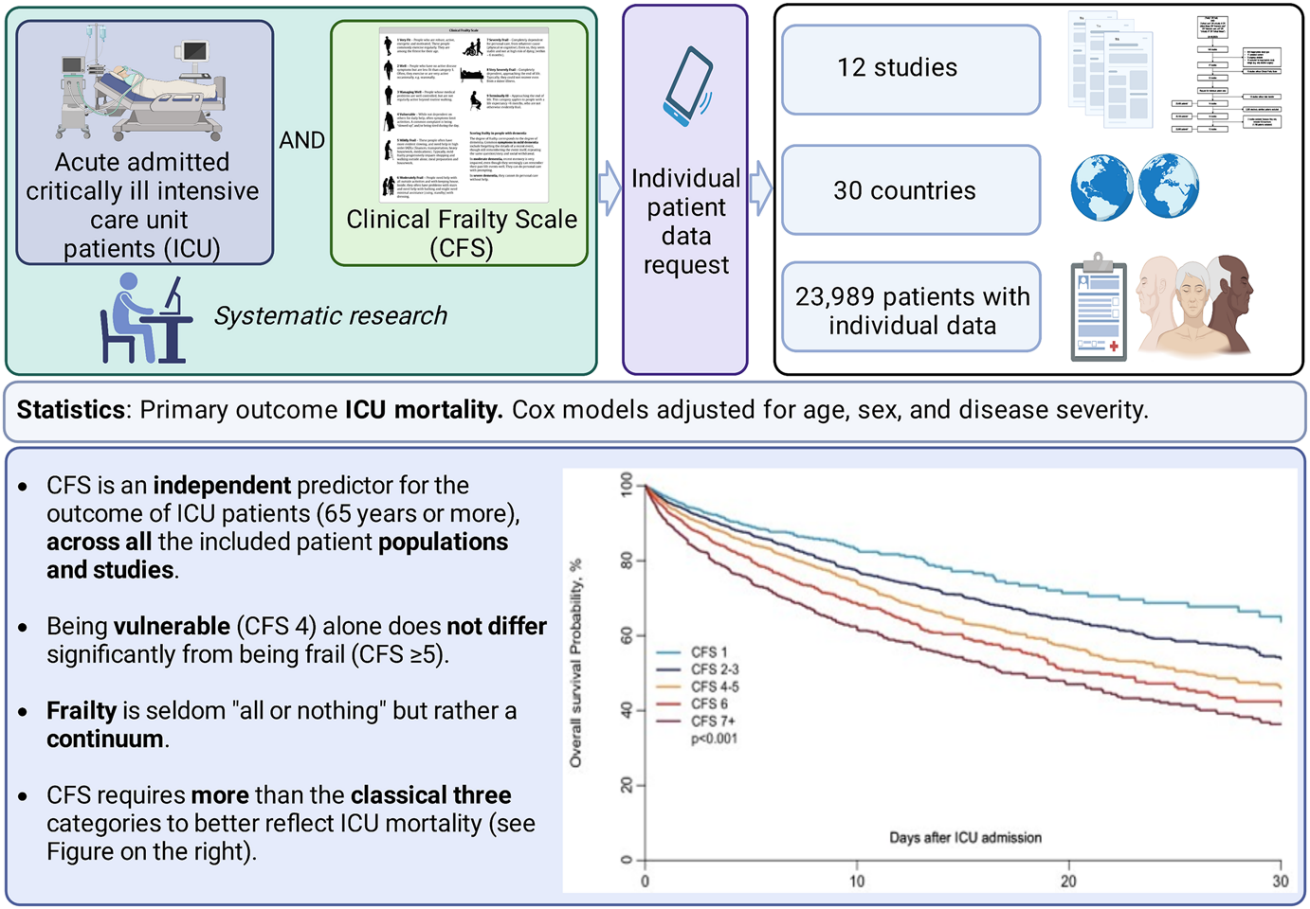

**Supplementary Information:**

The online version contains supplementary material available at 10.1186/s13613-023-01132-x.

## Background

Global life expectancy is increasing worldwide, leading to socio-demographic transition and affecting the entire medical field, especially intensive care medicine [1]. Demographic change increases the prevalence of multi-morbidity and results in a greater exposure to complex invasive procedures and interventions. Not all older ICU patients benefit from intensive care treatment [2], although the "relative benefit from an ICU admission" is higher in older than in younger patients [3]. In recent years, a significant increase in the number of old and very old intensive care patients has been observed in many countries [4], resulting in a greater relative proportion of health resources being utilised by this growing demographic group [5]. This is relevant as old patients are the fastest growing subgroup in intensive care medicine [6]. These old and very old patients have thus become a focus of research; it is now a consensus that chronological age alone is not a suitable criterion for assessing the prognosis of critically ill ICU patients [7]. Away from clinical trials, "ageism" is a common problem [8, 9]. For this reason, alternative concepts focusing on frailty rather than age alone have been developed. Frailty is a complex syndrome characterised by reduced physiological resistance against stressors. In most definitions, frailty is age-related [10], although some investigators also used this concept in younger patients [11]. In this context, Clinical Frailty Scale (CFS, see Additional file 1: Figure S1) is an established measurement and screening tool [12] in acute and critical care [13], which is easy to use [13], and that would later warrant a more detailed and comprehensive evaluation for confirmation. Accordingly, it has been tested in numerous studies in different contexts and offers an excellent inter-rater variability [14, 15]. Recently, numerous studies and meta-analyses have demonstrated that pre-acute frailty is predictive of outcome in critically ill patients with SARS-CoV-2 [16–20]. Both clinical trials [21–23] and meta-analyses [24] have assessed the value of CFS in predicting prognosis in ICU care. However, to date there is no systematic individual participant-level data meta-analysis that has pooled and evaluated individual data, to look at trans-national validity and generalisability, although every individual patient data meta-analysis depends on the quality and the selection of patients in all the individual studies. This gap will be filled by the present investigation, which includes several recently published large clinical studies, forming the largest database of individual patient data from multiple intensive care units.

Thus, this study will answer many urgent questions, in particular: is the CFS a valid and reproducible instrument through many different countries from the “western world” with different health care systems? Is the currently, most widely used classification (CFS 1–3; CFS 4 and CFS 5–8) useful for the prediction of ICU outcome? Is there a clinically relevant difference between “very fit” and “well” or between “vulnerable” and “frail”? This individual patient database can provide clarity and important new insights about these issues. In summary, the main objective to assess the relationship between ICU mortality as primary endpoint and frailty in all included patients. In the second step, we will repeat this analysis comparing older patients (≥ 65 years). Last, the widely used CFS classification will be compared regarding its power to discriminate “vulnerable” from “frail” patients, and the data will be explored for alternative CFS classifications.

## Methods

### Aim, design and setting of the study

The review protocol was prospectively registered on the database Open Science Framework (OSF: https://osf.io/8buwk/). The present study was conceived as an individual patient meta-analysis of observational data. We followed the Meta-Analysis of Observational Studies in Epidemiology (MOOSE) convention for study selection, collection of data, and analysis [25].

### Systematic search

A systematic search of electronic databases (PubMed, The Cochrane Library, MEDLINE, Clinicaltrials.gov) was conducted to identify original research articles published from the earliest available records up to and including 24th June 2020. In the meantime (30th September 2022), new studies had been published that could not be included (see Additional file 1:). Boolean search phrases included search terms relevant to frailty and intensive care following Muscedere et al. [24]. All study designs except for meta-analysis, narrative reviews, case reports, and editorials were included. Studies that constituted only subgroup analysis of other studies were excluded to avoid the use of duplicate patient data. Pilot searching (screening titles/abstracts/keywords/full texts) of previously known articles was used to identify relevant keywords for each search term. Keywords were combined within terms using the 'OR' operator, and the final search phrase was constructed by combining the search terms using the 'AND' operator: ("frailty" OR "frail") AND ("critical care" OR “critically ill” OR “critical illness” OR "intensive care" OR "intensive care unit").

### Study selection

The search was performed and checked by two independent reviewers (RRB and PHB). They evaluated the retrieved titles and abstracts of all articles to identify potentially relevant studies. In the next step, all studies without a documented CFS were excluded. After the checks by the two independent reviewers, all principal investigators of the relevant studies were contacted to obtain anonymised individual patient data. Inclusion and exclusion criteria for studies and patients are displayed in Additional file 1: Table S1.

### Data collection

The anonymised data were transferred to the University Hospital of Duesseldorf. All different file formats were converted into Excel-Sheets. All data were homogenised to a pre-defined standard (see Additional file 1: Figure S2).

### Inclusion and exclusion criteria

All patients who were electively admitted due to scheduled surgery or interventions were excluded. Thus, for the primary analysis, all acute admissions from the included databases were included. For ICU-survival analysis, all patients from studies that only included ICU survivors were excluded.

### Assessment of quality

To determine the risk of bias and thus the methodological quality of the included studies, the assessment QUIPS ("Quality In Prognosis Studies") was applied [24–26]. The assessment of the studies was performed by three independent investigators (LP, LJ, TZ) on 13^th^ June 2022.

### Statistical analysis

#### Description of patients' characteristics

Age categories were pre-specified. Patient baseline characteristics were analysed as frequencies and percentages for categorical variables and as medians and interquartile ranges (IQRs) for continuous variables. Age groups were stratified a priori into patients < 65 years and ≥ 65 years [7]. Comparisons between age groups were evaluated using the Wilcoxon test for continuous variables and the χ2 or Fisher exact test for categorical variables as appropriate.

#### CFS classifications

The three commonly used categories of frailty were applied: "fit" (CFS 1–3), "vulnerable" (recently changed to "living with very mild frailty" [27], CFS 4), and "frail" (CFS 5–8).

#### Outcomes of the study

The primary endpoint was ICU mortality. The overall crude survival after ICU admission was estimated by the Kaplan-Meier method and compared between CFS categories using a log-rank test. The proportion of patients alive at ICU discharge were also compared. To further study the impact of CFS on ICU survival, Cox models were fitted, including 1. only CFS, and 2. CFS and all variables included in the database and available at ICU admission, namely measures of illness severity, sex, and age. Since all severity indexes are already correlated, only one severity index was included in each model. The three different models were therefore as follows: one model used SOFA [28] as severity index, one SAPS II [29] as severity index, and one APACHE II [30] as severity index. We could not adjust for a centre-effect, because this data was not accessible for all included studies. Robust sandwich estimators to estimate the variance–covariance matrix of the regression coefficient estimates were used to account for the clustering of patients within studies.

#### Dealing with missing data

We first estimated our models on the complete data set of all patients with CFS and outcome data and then used multiple imputations for participants with missing data, using predictive mean matching for continuous variables, logistic regression for binary data, and polytomous regression for (unordered) categorical data. The cumulative baseline hazard was approximated by the Nelson–Aalen estimator and included in the imputation model and outcomes, all severity indexes, sex and age. Fifty imputations were drawn. Cox models were estimated in each imputed dataset, and estimates were combined using Rubin's rules to give an overall estimate of parameters and corresponding variance‐covariance matrix.

#### Subgroup comparison

The age threshold for inclusion varied across studies, so it was decided to repeat all analyses using an arbitral and literature-based cut-off of 65 years of age [7].

#### Statistical analysis

All tests were two-sided, and a p-value of < 0.05 was considered statistically significant. Statistical analyses were performed with R 3.2.3 software packages (R Development Core Team, Vienna, Austria). Additional file 1: Figure S3 provides an overview of the statistical approach.

## Results

### Study selection

**Fig. 1:**
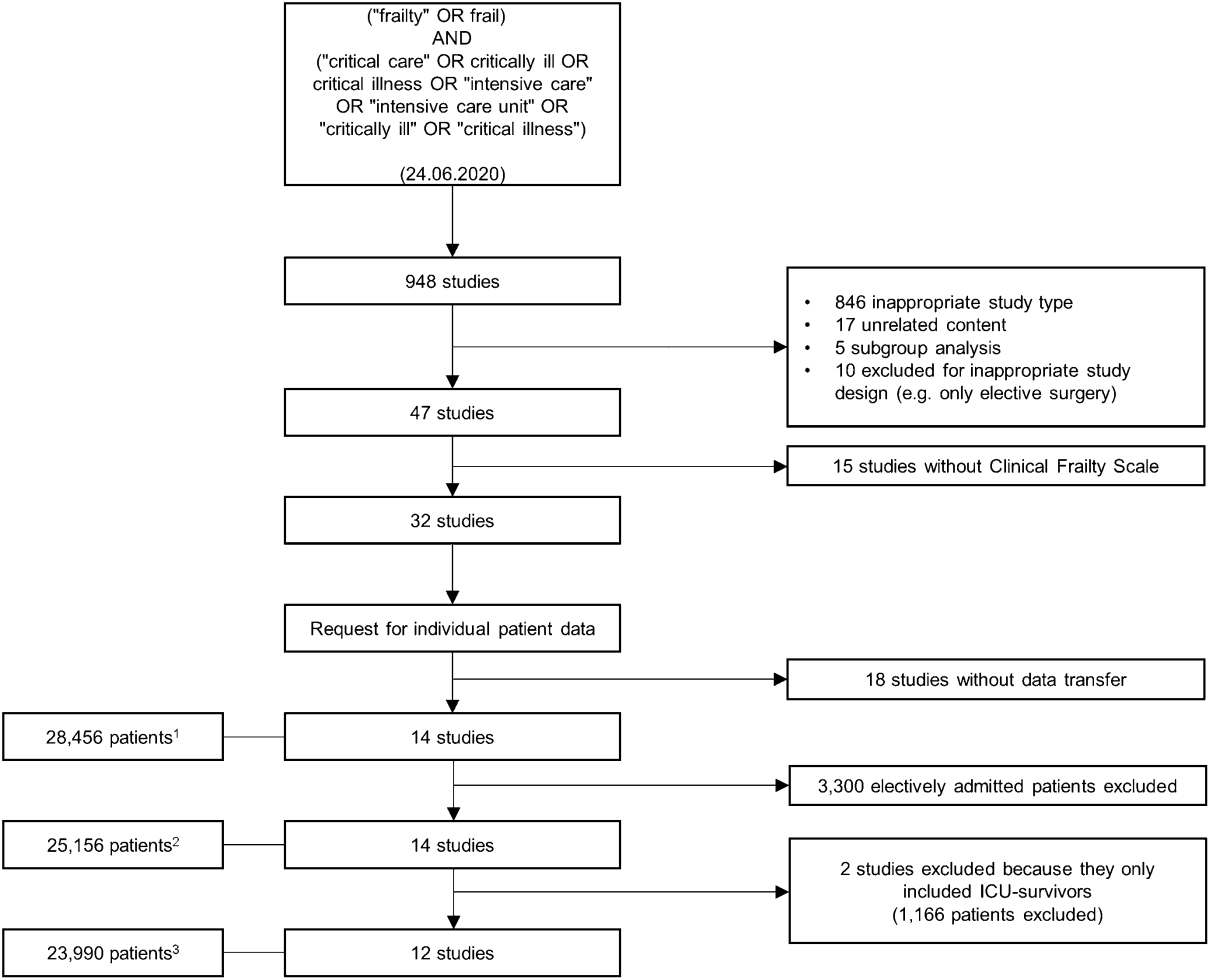
Consort diagram

Altogether, 948 studies were screened. According to the pre-defined criteria (see Additional file 1: Table S1), 901 studies were excluded. Of the remaining 47 studies, 32 used the CFS to screen for frailty (*n* = 59,341 patients). Investigators from 14 studies answered our invitation to join this project (*n* = 28,456 patients, see Fig. 1). Two studies had to be excluded because they only included ICU survivors [31, 32]. Thus, 12 studies, representing 30 different countries, were included in this meta-analysis, which after excluding patients who were electively admitted for scheduled surgery, consisted of 23,989 patients with individual data [22, 23, 33–42]. Additional file 1: Tables S2 and S3 summarise the main characteristics of the studies included. As the studies did not all study the same outcomes, Additional file 1: Table S4 gives an overview of what data was provided by each study.

### Quality

Most of the studies included evidenced a low overall risk of bias. Three studies had a moderate [33, 36, 38] and three a high risk of bias [31, 39, 41]. Additional file 1: Table S5 displays the detailed analysis of bias.

### Baseline characteristics at ICU admission

**Fig. 2:**
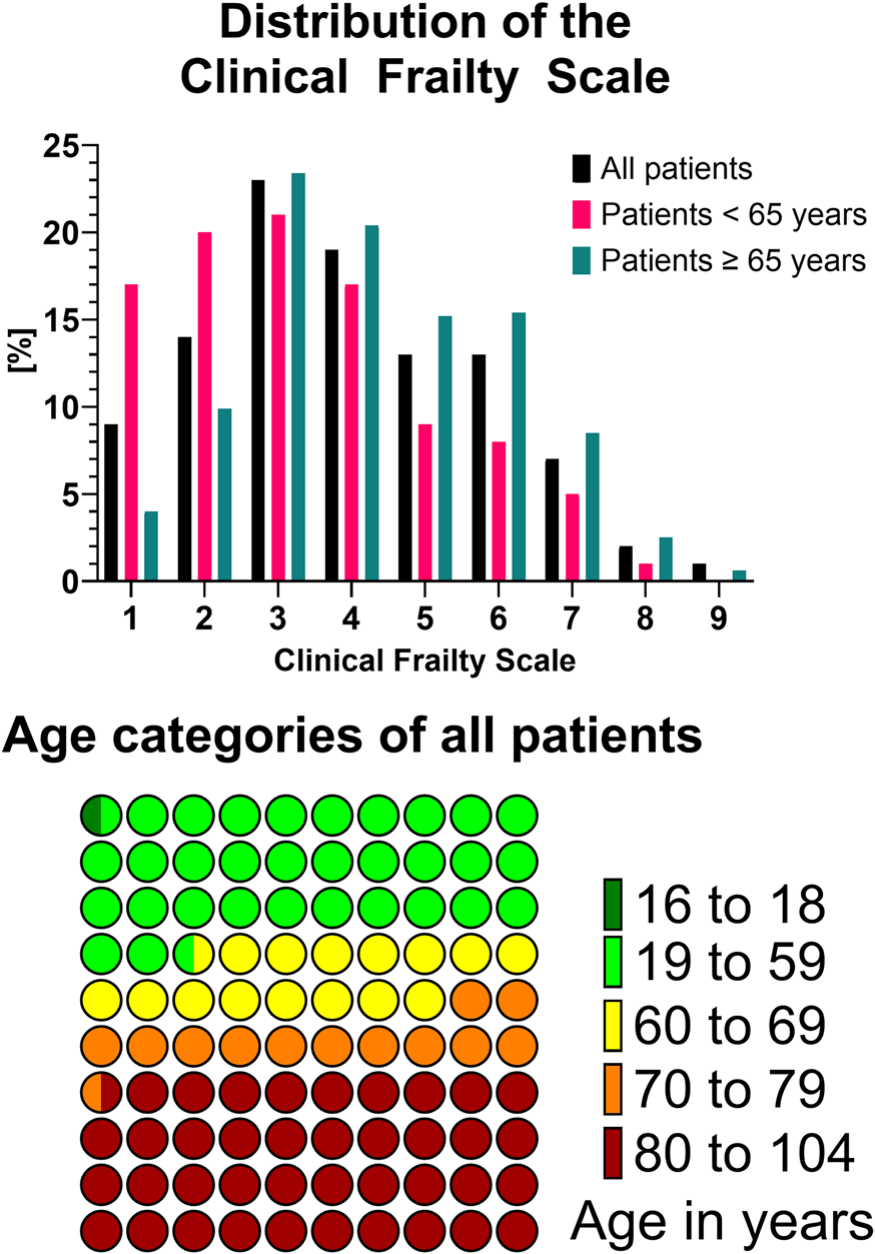
Distribution of CFS and age categories

Overall, there were more men than women (43.2% females and 56.8% males). The median age on admission was 71 years (IQR 55–82), but three studies included only patients who were 80 years or older [40, 45, 46], and two studies included patients older than 70 years [34, 35], and three studies patients older than 65 years [41, 42, 44]. Frailty was distributed normally (Fig. 2A), with most patients having a CFS of 3 ("managing well") before the acute illness. The distribution of age categories is depicted in Fig. 2B. On admission, the median SOFA was 7 (IQR4-10), SAPS II 50.5 (IQR 39–65.8), and APACHE II 20 (IQR 14–26, see Table 1).

**Table 1:** Patient characteristics on ICU admission

Characteristics on ICU admission	All patients	< 65 years	≥ 65 years	*P*-value
Patients	23,989	9633	14,356	
Age [years]	71 [55–82]	50 [37–58]	81 [75–85]	< 0.0001
SOFA	7 [4–10]	6 [3–9]	7 [4–10]	< 0.0001
SAPS II	50.5[8, 18, 39–53]	n/a2	50.5 [8, 18, 39–53]	< 0.0001
APACHE II	20 [14–26]	18 [12–24]	22 [17–28]	< 0.0001
Male gender	13,626 (56.8)	5669 (58.8)	7956 (55.4)	< 0.0001
Clinical frailty scale				< 0.0001
1	2006 (9.1)	1462 (17.3)	544 (4.0)	
2	3067 (13.9)	1714 (20.3)	1353 (9.9)	
3	4970 (22.5)	1775 (21)	3195 (23.4)	
4	4236 (19.2)	1445 (17.1)	2790 (20.4)	
5	2867 (13)	785 (9.3)	2082 (15.2)	
6	2819 (12.8)	711 (8.4)	2108 (15.4)	
7	1599 (7.2)	436 (5.2)	1163 (8.5)	
8	425 (1.9)	82 (1.0)	343 (2.5)	
9	109 (0.5)	33 (0.4)	76 (0.6)	
Clinical frailty scale (categories)				< 0.0001
fit (CFS 1–3)	10,043 (45.4)	4951 (58.6)	5092 (37.3)	
vulnerable (CFS 4)	4236 (19.2)	1445 (17.1)	2790 (20.4)	
frail (CFS 5–8)	7819 (35.4)	2047 (24.2)	5772 (42.3)	

Continuous variables: median [IQR]; categorial variables: Number (percentage); N = 23,991 patients; ^1^ 65–69 years; ^2^ Studies including SAPS II recruited only patients ≥ 65 years

### Intensive care treatment and outcome in all patients

Table 2 illustrates the intensive care treatment and outcome. During the ICU stay, most patients underwent mechanical ventilation (14,535 patients, 60.7%) for a median duration of one day (IQR 0–3.7 days), and most patients were receiving vasoactive drugs (12,329 patients, 53.3%). The median duration of vasoactive drugs was 0.25 days (IQR 0–2 days). Renal replacement therapy was received by 1,962 patients (8.1%). The majority of patients had missing information regarding treatment limitations (*n* = 16,536), but in those patients where the information was present, most patients did not have limitations in life-sustaining therapy (*n* = 5,846; 67.8%). Almost one-fifth of the patients died during their ICU stay (4,575 patients, 19.1%). The median time to death in ICU was three days (IQR 1–78.1 days). Additional file 1: Figure S4 illustrates the median length of stay, percentage of mechanical ventilation and vasopressors for every single study.

**Table 2:** Intensive care treatment during ICU stay and outcome

Intensive care treatment during ICU stay		All patients	< 65 years	≥ 65 years	P-value
Mechanical ventilation		14,535 (60.7)	6259 (65)	8276 (57.8)	< 0.0001
Duration of mechanical ventilation	[days]	0.93 [0–3.7]	0.7 [0–3]	1.3 [0–4.5]	< 0.0001
Vasoactive drugs		12,329 (53.3)	4200 (45.6)	8129 (58.4)	< 0.0001
Duration of vasoactive drugs	[days]	0.25 [0–2]	0 [0–1.2]	0.8 [0–2.8]	< 0.0001
Limitation of life sustaining therapy	Any limitation	2774 (32.2)	38 (9.2)	2736 (33.3)	< 0.0001
	No limitation	5846 (67.8)	376 (90.8)	5469 (66.7)	
Length of stay ICU	[days]	3.62 [1.7–7.5]	3.5 [1.7–7]	3.7 [1.7–7.9]	0.0032
Death on ICU		4575 (19.1)	1144 (11.9)	3431 (24)	< 0.0001
Discharged from ICU		19,368 (80.9)	8489 (88.1)	10,878 (76)	< 0.0001

Continuous variables: median [IQR]; categorial variables: Number (percentage)

### Univariate analysis and multivariate adjustment about for the impact of CFS on ICU survival

**Fig. 3:**
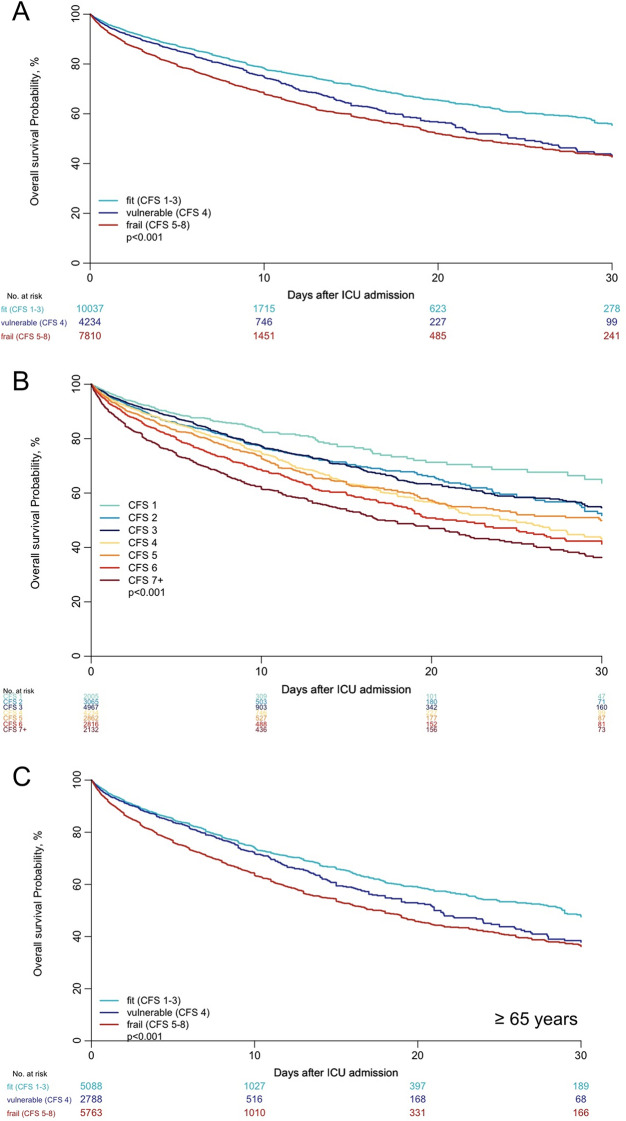
Kaplan–Meier for ICU mortality, patients stratified in three groups according to their CFS (**A**), and in seven groups according to their CFS (**B**), and in patients age 65 years or more according to their CFS (**C**)

Figure 3A shows the overall probability of ICU survival using the usual three categories of frailty. Figure 3B demonstrates the ICU survival across the seven CFS categories. SOFA was available in most studies (20,767 patients, 86.6%). Table 3 summarizes the regression analyses for ICU mortality including all patients. In complete case analysis, after adjustment for SOFA and other baseline covariates (age, gender), both "vulnerable" and "frail" patients had a significantly higher risk of ICU death when compared with "fit" patients (CFS 1–3). These results were confirmed using multiple imputations for "vulnerable" and "frail" patients compared to "fit" patients (Additional file 1: Tables S6-S7). Additional file 1: Table S8 collects the results for the adjustment for APACHE II and SAPS II. After adjustment for SAPS II (available in 2,256 patients, 9.4%) and the other covariates, being "frail" was significantly associated with increased risk of ICU death when using multiple imputations for patients with missing data, but not in the complete case analysis. When using SAPS II to adjust for severity, being "vulnerable" was not associated with an increased risk of ICU death compared to being fit. APACHE II was available in eight studies, including 14,086 patients (58.7%). CFS did not seem to impact ICU risk of death when adjusting for severity using APACHE II. Only three studies used APACHE III as a severity index. CFS was significantly associated with mortality when performing the same analysis using data from these three studies separately

**Table 3:** Regression analyses for ICU mortality including all patients

Complete case analysis—Model including only Frailty (n = 22,044)			
		HR^1^ (95%CI)	*P*-value
Frailty	vulnerable (CFS 4)	1.26 (1.11–1.43)	0.00024
	frail (CFS 5–8)	1.59 (1.53–1.66)	< 0.0001
Models including all variables available at baseline			
Complete Case Analysis—Model including SOFA as severity index (*n* = 20,767)			
		HR^a^ (95%CI)	P-value
Frailty	vulnerable (CFS 4)	1.07 (1.03–1.1)	0.00054
	frail (CFS 5–8)	1.26 (1.15–1.38)	< 0.0001
Severity	SOFA (one point increase)	1.17 (1.1–1.23)	< 0.0001
Gender	male vs female	0.97 (0.91–1.04)	0.3591
Age	Age (5 years increase)	1.11 (1.09–1.13)	< 0.0001
Multiple Imputation Analysis—Model including SOFA as severity index			
		HR^1^ (95%CI)	*P*-value
Frailty	vulnerable (CFS 4)	1.07 (1.03–1.11)	0.00139
	frail (CFS 5–8)	1.28 (1.15–1.41)	< 0.0001
Severity	SOFA (one point increase)	1.17 (1.11–1.23)	< 0.0001
Gender	male vs female	0.96 (0.9–1.02)	0.15900
Age	Age (5 years increase)	1.11 (1.09–1.13)	< 0.0001

^a^Reference "fit" defined as CFS score 1–3

### Comparison of patients' and ICU stays' characteristics in ICU patients above and below 65 years old

More than 50% of patients were above 65 years old at the time of ICU admission (Fig. 1). The percentage of male patients was significantly higher in older patients (55.4% versus 58.8%, *p* < 0.001). Older patients were more severely ill in terms of SAPS II, APACHE II, and SOFA scores as measures for illness severity at ICU admission (Table 1). Older patients had higher CFS scores than younger patients (CFS 5–8: 24.2% in patients below 65 versus 42.3% in patients above 65, Fig. 2). During the ICU stay, there were significant differences between older and younger patients in several procedures. Older patients received mechanical ventilation less often, but for a longer median duration. Furthermore, older patients received vasoactive drugs more frequently, for a longer median duration. Older patients had more limitations in life-sustaining therapy. Lengths of stay in ICU and hospital were longer for older patients. Crude ICU mortality was higher in older patients (3431 patients (24%) versus 1144 patients (11.9%), *p* < 0.0001), and their median time to death in ICU was 0.5 days shorter (3.1 days [1–8.8] versus 2.6 days [0.9–6.6], *p* < 0.0001). Older patients differed from younger patients regarding their baseline characteristics, course and life-sustaining therapies received in ICU. For this subgroup analysis, 13,602 patients aged 65 years or older were included. In the univariate analysis, "frail" (CFS ≥ 5) patients had an increased risk of ICU death as compared to fit patients (CFS 1–3, see Table 4). After adjustment for SOFA score, sex and age, "frail" patients had an increased risk of ICU death compared to fit patients. These results were confirmed in the complete case analysis when using multiple imputations for patients with missing information. Similarly, when using SAPS II or APACHE II to adjust for severity, "frail" patients showed an increased risk of ICU death compared to fit patients (Additional file 1: Table S6). By contrast, being "vulnerable" (CFS 4) was borderline significant or not-significant across all the analyses—depending on the used acute illness severity score (compared with the reference CFS of 1–3, see Additional file 1: Table S7).

**Table 4:** Regression analyses for ICU mortality including patients ≥ 65 years using a model including all variables available at baseline

Complete Case Analysis—Model including only frailty (*n* = 13,602)			
	HR^a^ (95%CI)	P-value	
vulnerable (CFS 4)	1.15 (1.07–1.24)	0.00021	
frail (CFS 5–8)	1.52 (1.43–1.62)	< 0.0001	
Complete Case Analysis—Model including SOFA as severity index (*n* = 12,972)			
		HR^a^ (95%CI)	P-value
Frailty	vulnerable (CFS 4)	1.1 (1.05–1.16)	0.00029
	frail (CFS 5–8)	1.34 (1.25–1.44)	< 0.0001
Severity	SOFA (one point increase)	1.14 (1.1–1.19)	< 0.0001
Gender	male vs female	0.97 (0.89–1.06)	0.51997
Age	Age (5 years increase)	1.2 (1.15–1.25)	< 0.0001
Multiple Imputation Analysis—Model including SOFA as severity index			
		HR^a^ (95%CI)	P-value
Frailty	vulnerable (CFS 4)	1.1 (1.04–1.17)	0.0006
	frail (CFS 5–8)	1.35 (1.26–1.45)	< 0.0001
Severity	SOFA (one point increase)	1.14 (1.1–1.19)	< 0.0001
Gender	male vs female	0.95 (0.87–1.04)	0.27293
Age	Age (5 years increase)	1.19 (1.15–1.23)	< 0.0001

^a^Reference: "fit" defined as CFS score 1–3

### Risk stratification using a different five-steps CFS categorisation

**Fig. 4:**
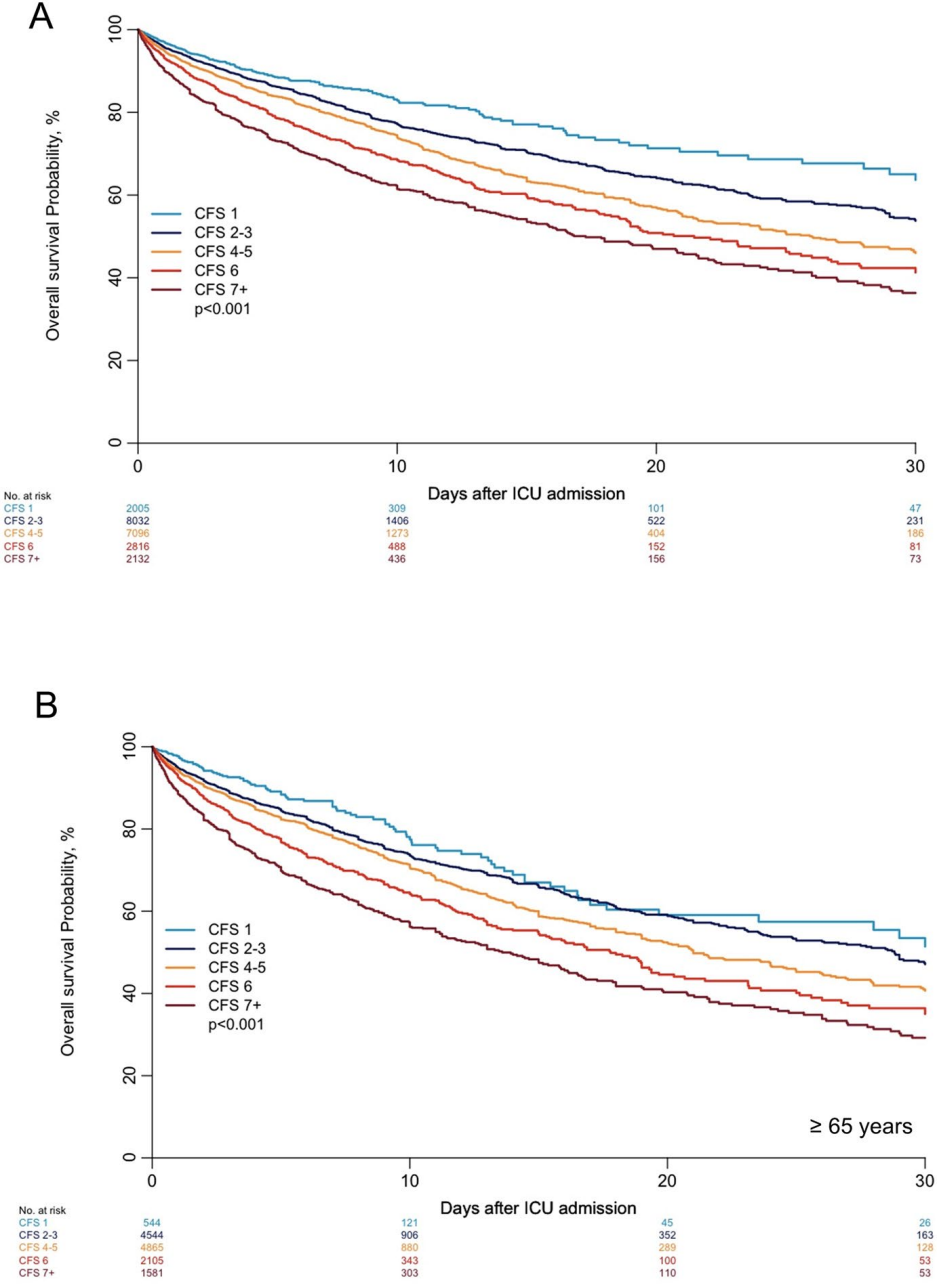
Kaplan–Meier for ICU mortality, all patients (**A**) and patients age 65 years or older (**B**) stratified in five groups according to their CFS

Based on this data exploration and visual examination of the unadjusted survival curves, a different classification was applied to reflect the impact of CFS on ICU outcome better (Fig. 4A): CFS 1 ("very fit") with the best ICU prognosis, CFS 2–3, then CFS 4–5, CFS 6, and worse than CFS 6). These observations were reproducible in patients aged 65 years or older (Fig. 4B). Accordingly, in the unadjusted Cox regression analysis," vulnerable" patients (CFS 4) and" frail" patients (defined as a CFS of 5–8) had a higher hazard of death compared with "fit" patients (CFS 1–3). Furthermore, patients with a CFS 2–3, 4–5, 6, 7 or more were associated with an increased risk of ICU death compared with patients with a CFS 1. In a complete case analysis after adjustment for SOFA, sex and age, HR for risk of ICU death for the alternative classification of CFS is displayed in Table 5. After adjustment, patients with a CFS of 4–5, a CFS of 6, and a CFS of 7 or worse evidenced a significantly increased risk for ICU mortality compared to “fit” patients.

**Table 5:** Complete case analysis after adjustment for SOFA, sex and age, HR for risk of ICU death for new classification of CFS

All patients	aHR	*P*-value
CFS 2–3 vs CFS 1	0.94 (0.71–1.24)	0.65311
CFS 4–5 vs CFS 1	1.01 (0.75–1.36)	0.95538
CFS 6 vs CFS 1	1.2 (0.91–1.57)	0.189
CFS 7 + vs CFS 1	1.43 (1.1–1.86)	0.00842
Patients ≥ 65 years	aHR	*P*-value
CFS 2–3 vs CFS 1	1.16 (0.94–1.43)	0.15898
CFS 4–5 vs CFS 1	1.28 (1.06–1.55)	0.00995
CFS 6 vs CFS 1	1.56 (1.32–1.84)	< 0.001
CFS 7 + vs CFS 1	1.78 (1.49–2.12)	< 0.001

## Discussion

The present study is based on the largest individual patient meta-analysis to date, with datasets from 23,989 acutely admitted ICU patients, and underlines the significant impact of being frail on ICU mortality—regardless their age. These patients were recruited into 12 different studies in 30 different countries with diverse health care systems. First, focusing on short-term outcomes, with this dataset it was possible to confirm that CFS is an independent prognostic factor in ≥ 65 years old patients even after adjustment for relevant co-factors. However, CFS does not consistently show an association with ICU mortality in patients younger than 65 years. Second, being "vulnerable" (CFS 4, according to the old nomenclature) as category on its own does not provide prognostic information about ICU mortality compared with being "frail", although there seems to be a relevance for this category regarding long-term outcome [21]. Third, considering that frailty is a continuum, using CFS with different severity stages, with an alternative classification might be proposed to reflect the incremental risk of mortality better. This must of course be validated in additional studies.

The most common classification of frailty using the CFS consists of "non-frail" patients (CFS 1–3), "vulnerable" patients (CFS 4), and "frail" patients (CFS 5–8). These stages suggest the existence of a group with low, intermediate, and high risk of adverse outcome, and they were proposed—for example—for early risk stratification and screening, although the “gold standard” to diagnose frailty remails a comprehensive geriatric assessment performed by geriatric medicine.

The original CFS from the Canadian Study of Health and Aging consisted of seven stages [43]. Later, two additional stages were added (8: very severely frail and 9: terminally ill) [27]. The WHO defines frailty as a clinically visible state in which the capability of older persons to deal with both routine and acute stressors is diminished. Higher age is often correlated with but is not synonymous with frailty [13]. The cumulative cellular and molecular impairment caused by genetic, epigenetic, and environmental causes lead to a reduced physiological reserve, potentially affecting all organ systems [13]. These aging processes vary in speed of progress and might occur over decades, resulting in considerable biological and functional heterogeneity among older patients [44].

Increasingly, CFS is also widely used in younger patients, although it was not developed for this purpose. It is here that this patient database can provide clarity. In our analysis, after adjustment for confounders, only a CFS of 7–9 was independently associated with ICU mortality in patients younger than 65, although in older patients, all stages from a CFS of 4–5 upwards had a significant relationship. This difference may be clinically sensible, because younger patients, even if they are frail (CFS 4, 5, 6), might still survive their ICU admission.

Regarding the interaction between CFS and scores for acute illness, we noticed that in patients aged 65 years or older, a CFS of 4 is more significantly associated with outcome when adjusting for SOFA but not after adjusting for SAPS II or APACHE II. Additional file 1: Table S9 illustrates the differences between these scores. The reason might be that adjusting for these two severity scores might lead to overfitting as they both include pre-existing chronic diseases and age.

Regarding patients suffering from COVID-19, Kastora et al. recently performed a meta-analysis including 34 prospective and retrospective cohort studies focusing on CFS and COVID-19 mortality [45]. They classified CFS 1–3 for patients with a lower risk, CFS 4–5 as moderate risk, and CFS 6–9 as high risk for COVID-19 mortality. By contrast, Darvall et al. used four different categories in their secondary analysis of data from a prospective cohort study including 269,785 critically ill adults from 168 ICUs in Australia and New Zealand CFS 1–2, 3–4, 5–6, 7–8, indicating that CFS has previously been divided in different ways [46].

Some additional surprising new insights can be gained from this metanalysis that might generate hypotheses for further research. In the Kaplan-Meier analysis, patients with a CFS of 4 have a similar ICU survival as patients with a CFS of 5. Thus, there seems to be an overlap regarding the prognosis between patients with CFS 4 and 5. Furthermore, patients with a CFS of 4 do not appear to differ significantly from those with a CFS of 5–8. By contrast, our various regression analyses failed to find an independent association of a CFS of 4 with ICU mortality, depending on the adjustment, with the overall adjustment for APACHE II resulting in CFS no longer being independently associated with the primary endpoint. Incidentally, this observation remained constant across studies in the subgroup analyses. Another interesting finding from this data is that patients with a CFS of 1 appear to have a significantly better probability of ICU survival than patients with a CFS of 2–3. Notably, 2005 patients were reported to have a CFS of 1 (approximately 8% of the total study population). This emphasizes that physicians, relatives, and patients might overestimate their patient's fitness. One possible reason could be their fear of denial of ICU admittance in case of a lower CFS. In future studies, this overestimation could be prevented by using a more systematic approach to CFS estimation, for example, by adding a decision tree as recently proposed by Theo et al.[47]. Another important aspect for future studies might be investigating not only short-term mortality but also long-term functional mortality as an important clinical endpoint in intensive care medicine.

## Limitations

This meta-analysis has some limitations. First, all included studies focused on patients who were admitted to the ICU. This—commonly occurring—inclusion criterion of ICU admission leads to a selection bias, because frail patients whose ICU admission was denied cannot be analysed. Thus, there is an unknown percentage of frail (older) patients with acute illness that have been “rejected” for ICU therapy. As very in individual patients’ data meta-analysis, we rely on the active cooperation with the researchers who provide their original datasets. In fact, only some of the identified investigators provided data. Next, the data sets were as heterogeneous as their underlying study design. None of the included studies collected exactly the same variables. In consequence, we chose those endpoints with the highest degree of data completeness. For in instance, choosing (short term) mortality as primary endpoint in intensive medicine bears several problems although ICU mortality one of the most often used outcome [48].. 30-day mortality instead would not have solved all methodological problems, although it is supposed to be less dependent upon discharge policies. In fact, older frail patients might die later (i.e., in hospital or in the first 3–6 months) or they might be discharged from the ICU with a limitation for life-sustaining therapy and thus not be suitable for readmission. In addition, there is rising evidence that frailty continues to modify risk of death and morbidity long after ICU admission. Thus, many longer-term mortality and morbidity questions (i.e., health-related quality of life, disability, and institutionalisation) could not be elucidated. For this individual per-patient level meta-analysis, we chose ICU mortality as primary endpoint because there were significantly less missing fields compared to 30-days mortality. Another important issue in this context is the occurrence of limitations for life-sustaining therapy. In fact, these limitations represent a crucial confounder when investigating the impact of frailty on mortality that has not been reported equally in all included studies leading to a higher number of missing fields for this information (15,370 missing values). In sum, this individual per-patient level meta-analysis could only use data as collected before. Another issue is that in the present analysis, anchoring of the assessment of frailty in the ICU was also not captured, and this may represent an unrecorded competing risk. Methods for recording CFS vary across the studies, and the assessment of recording categories rather than a continuous variable was also inconsistent. In addition, frailty assessment was performed at ICU admission. In 2020 / 2021, many studies investigating the impact of frailty on disease risk and severity in SARS-CoV-2 (COVID-19) infection have also appeared [20, 49–52]. The present meta-analysis did not include patients with COVID-19, as the data were not available at the time of the search. Furthermore, the high number of patients with primary COVID-19 would have been over represented and thus potentially skewed the data. This systematic search of electronic databases was conducted until 24th June 2020, however in the interim, only few new eligible studies have emerged (30th September 2022, see Electronic Supplement). The distribution of CFS values was different in patients below and above 65 years old, and there was only a small number of older patients with a CFS of 1. Another problem was that some studies did not report SOFA. Regarding covariates, "gender" was not specifically captured in the included studies, but should normally represent the biological sex. Sex constitutes an important variable—but the influence of gender role in prognosis is difficult to assess, and there are differences that are important to measure [53]. Another relevant limitation is using the arbitral age cut-off of 65 years. In fact, many studies prefer 80 years or older to define “older” patients. Furthermore, some of the included study did only include older (> 65 years) or old (> 80 Years), which contributes to a further selection-bias when merging the databases. However, the ability to analyse patients of different age categories within one database could be considered as strength. On the other hand, it is questionable if the global concept of frailty should be equally applied in younger patients [21, 28]. In fact, a recent metanalysis by Spiers et al. found only limited evidence that frailty predicts the outcome with a sufficient validity in younger populations [11]. Last, the proposed “alternative” classification had not been a priori defined as an analysis and has not been validated to date.

## Conclusion

CFS is a valid and reproducible marker for early risk stratification of critically ill patients across a number of studies including patients from 30 countries from five continents (Additional file 1: Table S10). In older ICU-patients, being frail is an independent risk factor for increased mortality, regardless of the acute underlying disease leading to admission. Very fit patients (CFS 1) have a significantly better prognosis than all other patients. There seems to be an important overlap between a CFS of 4 and 5 ("mildly frail" and "moderately frail"). Measuring across the full spectrum of the CFS rather than grouping into categories better reflects the ICU outcome. Future randomised prospective studies should examine the extent to which early staging of the CFS can trigger interventions that improve outcome – or situations in which it might be better, not to intervene. CFS could be used in patients younger than 65 years, but the cut-off would be different from older patients.

## Supplementary Information

**Additional file 1: Figure S1**. Clinical Frailty Scale. Permission to use this scale was granted from Dalhousie University, Ca, May 15 2017. **Figure S2**. Flow chart showing the collection, conversion, extraction, integration, and control of the individual patient data. **Figure S3**. Overview on the statistical approach. **Table S1**. Inclusion and exclusion criteria for studies and patients, respectively. **Table S2**. Overview about the studies that contributed data – Part 1. **Table S3**. Overview about the studies that contributed data – Part 2. **Table S4**. Reported and collected data for each study. **Table S5**. Quality rating for the risk of bias using QUIPS. **Table S6**. aHRfor being frail. **Table S7**. aHRfor being vulnerable. **Table S8**. Regression analyses for ICU mortality, adjusted to APACHE II or SAPS II. **Table S9**. Overview on the different ICU-scores SOFA; SAPS II and APACHE II. **Table S10**. Origin countries of the included data sets.

## Data Availability

Individual participant data that underlie the results reported in this article are not available because all datasets were received confidentially. The anonymised data can be requested from each investigator if required.
