# Supplementary material for: The Clinical Frailty Scale for mortality prediction of old acutely admitted intensive care patients: a meta-analysis of individual patient-level data
Source: Ann Intensive Care. 2023 May 3;13:37. doi: 10.1186/s13613-023-01132-x (PMC10155148; doi:10.1186/s13613-023-01132-x)
Supplement: Supplementary file 1 — Additional file 1: Figure S1. Clinical Frailty Scale. Permission to use this scale was granted from Dalhousie University, Ca, May 15 2017. Figure S2. Flow chart showing the collection, conversion, extraction, integration, and control of the individual patient data. Figure S3. Overview on the statistical approach. Table S1. Inclusion and exclusion criteria for studies and patients, respectively. Table S2. Overview about the studies that contributed data – Part 1. Table S3. Overview about the studies that contributed data – Part 2. Table S4. Reported and collected data for each study. Table S5. Quality rating for the risk of bias using QUIPS. Table S6. aHRfor being frail. Table S7. aHRfor being vulnerable. Table S8. Regression analyses for ICU mortality, adjusted to APACHE II or SAPS II. Table S9. Overview on the different ICU-scores SOFA; SAPS II and APACHE II. Table S10. Origin countries of the included data sets. [file 13613_2023_1132_MOESM1_ESM.docx]

**Additional figures**


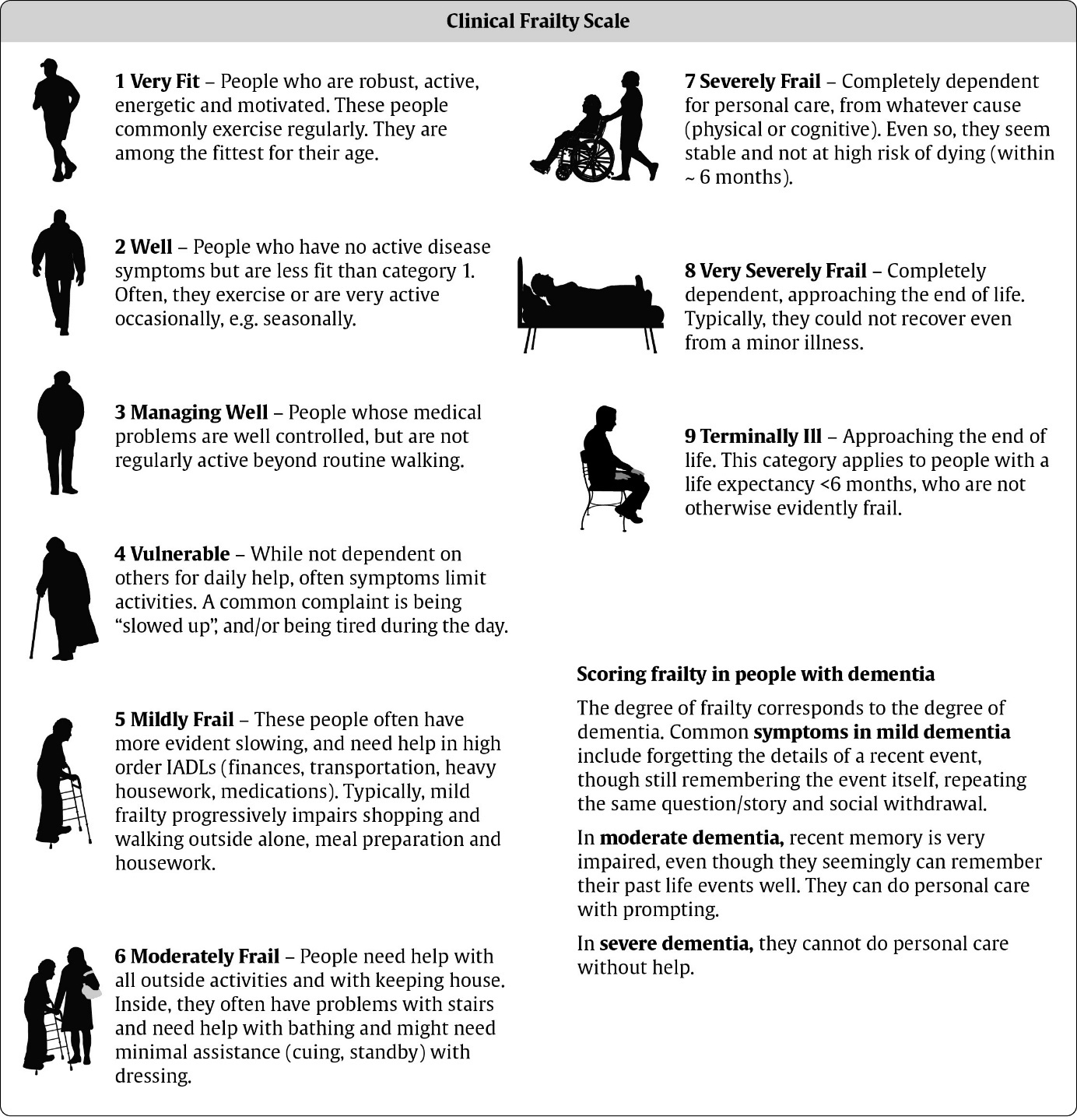


**Additional file 1: Figure S1:** Clinical Frailty Scale (CFS). Permission to use this scale was granted from Dalhousie University, Ca, May 15 2017.


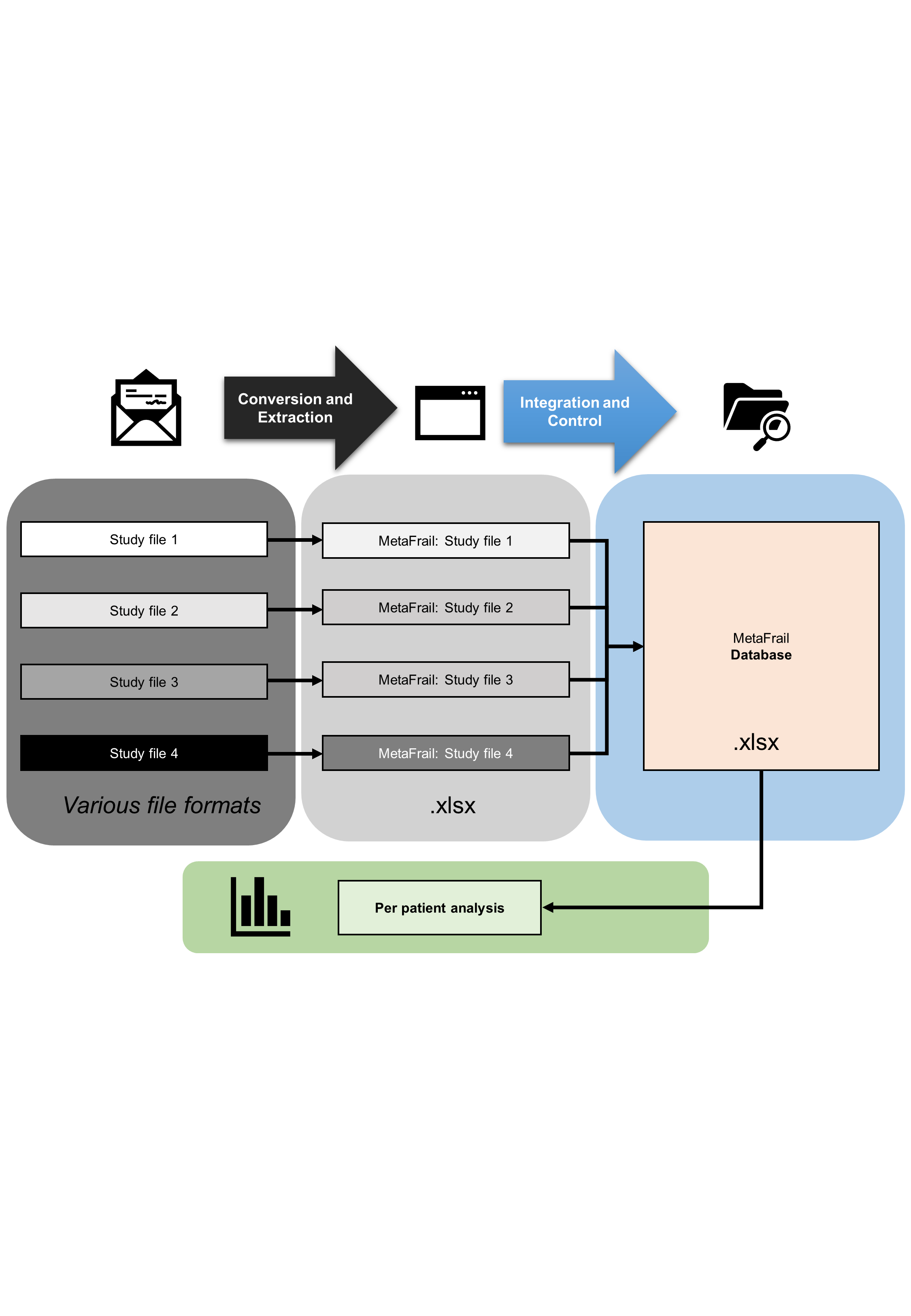


**Additional file 1: Figure S2:** Flow chart showing the collection, conversion, extraction, integration, and control of the individual patient data

**
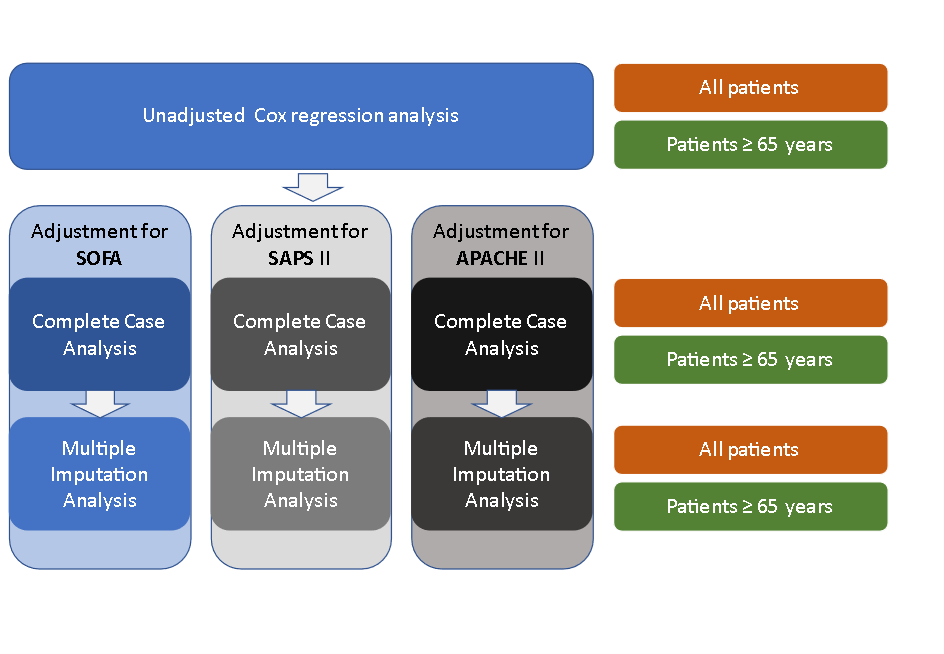
 Additional file 1: Figure S3:** Overview on the statistical approach


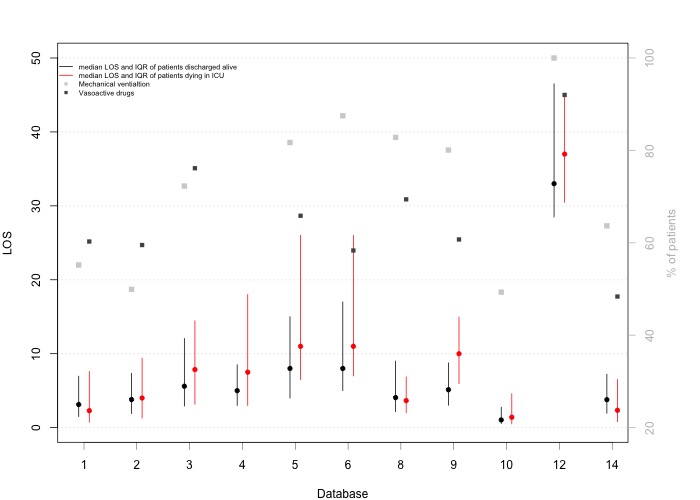


**Additional file 1: Figure S4:** Median length of stay (with IQR) for patients who were discharged alive from the ICU (black) and patients who died on the ICU (red) [days], including the percentage of patients receiving mechanical ventilation (grey dots) and/or vasoactive drugs (black dots) [%].

**Additional file 1: tables**

| **Pre-defined inclusion criteria for the screening process** | **Pre-defined exclusion criteria after the screening process** |
| --- | --- |
| - Frailty must be assessed. - Clinical Frailty Scale must have been assessed - Included patients must have been admitted to an intensive care unit - If there are multiple subgroup analyses of a study, then only the main study will be included (Each patient is included only once) - appropriate study type (observational, retrospective, or prospective study) - related content (frailty and intensive care unit) - availability of anonymized individual patient data | - Electively admitted patients |

**Additional file 1: Table S1:** Inclusion and exclusion criteria for studies and patients, respectively

| **Number** | **First Author** | **Title** | **DOI** | **patients** | **country** | **year** |
| --- | --- | --- | --- | --- | --- | --- |
| 1 | Flaatten | The impact of frailty on ICU and 30-day mortality and the level of care in very elderly patients (≥ 80 years) | 10.1007/s00134-017-4940-8 | 5021 | 21 European countries | 2017 |
| 2 | Guidet | The contribution of frailty, cognition, activity of daily life and comorbidities on outcome in acutely admitted patients over 80 years in European ICUs: the VIP2 study | 10.1007/s00134-019-05853-1 | 3920 | 22 countries | 2020 |
| 3 | Silva-Obregón | Frailty as a predictor of short- and long-term mortality in critically ill older medical patients | 10.1016/j.jcrc.2019.10.018 | 285 | Spain | 2020 |
| 4 | Ruiz de Gopegui Miguelena | Influence of frailty in the outcome of surgical patients over 70 years old with admission criteria in ICU | 10.1016/j.ciresp.2020.04.022 | 90 | Spain | 2020 |
| 5 | Langlais | Does the clinical frailty score improve the accuracy of the SOFA score in predicting hospital mortality in elderly critically ill patients? A prospective observational study | 10.1016/j.jcrc.2018.04.012 | 189 | France | 2018 |
| 6 | Le Maguet | Prevalence and impact of frailty on mortality in elderly ICU patients: a prospective, multicenter, observational study | 10.1007/s00134-014-3253-4 | 196 | France | 2014 |
| 7 | Darvall | Contributors to frailty in critical illness: multidimensional analysis of the Clinical Frailty Scale | 10.1016/j.jcrc.2019.04.032 | 160 | Australia | 2019 |
| 8 | Hewitt | The FRAIL-FIT study: Frailty's relationship with adverse-event incidence in the longer term, at one year following intensive care unit treatment - A retrospective observational cohort study | 10.1177/1751143719838212 | 400 | UK | 2020 |
| 9 | Heyland | Recovery after critical illness in patients aged 80 years or older: a multi-center prospective observational cohort study | 10.1007/s00134-015-4028-2 | 610 | Canada | 2015 |
| 10 | De Geer | Frailty predicts 30-day mortality in intensive care patients: A prospective prediction study | 10.1097/eja.0000000000001156 | 872 | Sweden | 2020 |
| 11 | Geense | Changes in frailty among ICU survivors and associated factors: Results of a one-year prospective cohort study using the Dutch Clinical Frailty Scale | 10.1016/j.jcrc.2019.10.016 | 1300 | Netherlands | 2020 |
| 12 | Papageorgiou | Frailty in elderly ICU patients in Greece: a prospective, observational study | 10.21037/atm.2018.02.03 | 36 | Greece | 2018 |
| 13 | Hope | Frailty, Acute Organ Dysfunction, and Increased Disability After Hospitalization in Older Adults Who Survive Critical Illness: A Prospective Cohort Study | 10.1177/0885066619881115 | 302 | USA | 2019 |
| 14 | Montgomery | Implementation of population-level screening for frailty among patients admitted to adult intensive care in Alberta, Canada | 10.1007/s12630-019-01414-8 | 15238 | Canada | 2019 |

**Additional file 1: Table S2:** Overview about the studies that contributed data – Part 1

| **Number** | **Study design** | **Inclusion criteria** | **Primary outcome** |
| --- | --- | --- | --- |
| 1 | transnational prospective cohort study | first consecutive 20 very old (≥ 80 years) patients admitted to the ICU within a 3-month inclusion period | ICU and 30-day mortality and survival at 30 days |
| 2 | Prospective cohort study with 242 ICUs | Age ≥80 years, admitted to ICU (May 2018 – May 2019) | 30-day survival |
| 3 | retrospective cohort study | Age ≥70 years, admitted to ICU (2009-2017) | ICU, hospital and 30-day mortality as short-term, and 3-, 6- and 12-month mortality as long-term mortality |
| 4 | prospective and observational study of ICU patients of a third level hospital | Age ≥70 years, admitted to ICU after elective or emergent surgical intervention on digestive system (June 1,2018-June 1,2019) | Six months mortality |
| 5 | prospective observational study | aged ≥65 years and hospitalized ≥24 h in ICU | Hospital mortality |
| 6 | prospective, multicenter, observational study | age ≥65 years hospitalized for >24 h during a 6-month study period | ICU mortality |
| 7 | prospective cohort study | aged ≥50 years admitted between February-June 2017 | Comparison of (CFS) with the Edmonton Frail Scale (EFS) |
| 8 | single-centre retrospective observational cohort study | ICU patients | ICU, hospital and one-year mortality |
| 9 | Prospective, longitudinal cohort study | Age ≥ 80, medical or urgent surgical diagnosis, ICU admittance ≥24 h | ICU, hospital and one-year mortality |
| 10 | prospective study with a comparison of two prediction models | tertiary mixed ICU, from January 2017 to June 2018 | 30-days mortality |
| 11 | Prospective cohort multicenter study (from MONITOR-IC study) | Patients 16 years or older, expected to survive the ICU, and admitted for at least 12 h to the ICU | Frailty after 12 months |
| 12 | prospective, observational study | Aged ≥65 years (1 year span) | ICU, hospital, and six-month mortality |
| 13 | prospective observational cohort study | survived critical illness | Activities of Daily Living disability levels through to 6 months after discharge |
| 14 | Retrospective cohort study of adult admissions to 17 ICUs | patients admitted to adult intensive care | Hospital mortality |

**Additional file 1: Table S3:** Overview about the studies that contributed data – Part 2

| **Study** | **1** | **2** | **6** | **5** | **3** | **7** | **4** | **9** | **8** | **11** | **10** | **12** | **13** | **14** |
| --- | --- | --- | --- | --- | --- | --- | --- | --- | --- | --- | --- | --- | --- | --- |
| **Age** | x | x | x | x | x | x | x | x | x | x | x | x | x | x |
| **Sex** | x | x | x | x | x | x | x | x | x | x | x | x | x | x |
| **Type of Admission** | x |  | x | x | x |  |  | x | x | x | x | x | x | x |
| **Apache II** | x |  |  |  | x |  | x | x | x | x |  | x |  | x |
| **Saps2** | x |  | x | x | x | x |  |  |  | x | x | x |  |  |
| **SAPSIII** |  |  |  |  |  |  |  |  |  |  | x |  |  |  |
| **APACHE IV** |  |  |  |  |  |  |  |  |  |  |  |  | x |  |
| **Clinical Frailty Scale (CFS)** | x | x | x | x | x | x | x | x | x | x | x | x | x | x |
| **SOFA** | x | x | x | x | x |  |  | x |  |  |  | x | x | x |
| **Mechanical ventilation (MV)** | x | x | x | x | x | x |  | x | x | x | x | x | x | x |
| **Duration of MV** |  | x | x | x | x |  |  | x | x |  |  | x | x | x |
| **Vasoactive drugs (VD)** | x | x | x | x | x | x |  | x | x | x |  | x | x | x |
| **Duration of VD** |  | x |  |  | x |  |  | x | x |  |  |  |  | x |
| **Renal replacement therapy (RRT)** | x | x | x | x | x | x |  | x | x |  | x |  | x | x |
| **Duration of RRT** |  | x |  |  | x |  |  | x | x |  |  |  |  | x |
| **Length of stay ICU** | x | x | x | x | x | x | x |  | x | x | x | x | x | x |
| **Length of stay hospital** |  |  | x | x | x | x | x | x | x |  | x |  | x | x |
| **Any limitation in life-sustaining therapy** | x | x |  |  |  | x |  | x |  |  | x |  |  |  |
| **Death on the ICU** | x | x | x | x | x | x | x | x | x | x | x | x | x | x |
| **Death in the hospital** |  |  | x | x | x | x | x | x | x |  |  | x | x | x |
| **Death after 30 days** |  |  |  |  |  |  |  |  |  |  |  | x | x |  |
| **Death after six months** |  | x | x | x |  | x | x | x | x |  | x | x |  |  |

**Additional file 1: Table S4:** Reported and collected data for each study

|  | Study participation | Study attrition | Prognostic factor measurement | Outcome measurement | Study confounding | Statistical analysis and reporting | **Overall Risk of Bias** |
| --- | --- | --- | --- | --- | --- | --- | --- |
| *Flaatten 2017* |  |  |  |  |  |  |  |
| *Guidet 2019* |  |  |  |  |  |  |  |
| *Silva-Obrégon 2019* |  |  |  |  |  |  |  |
| *Ruiz 2020* |  |  |  |  |  |  |  |
| *Langlais 2018* |  |  |  |  |  |  |  |
| *Le Maguet 2014* |  |  |  |  |  |  |  |
| *Darvall 2019* |  |  |  |  |  |  |  |
| *Hewitt 2019* |  |  |  |  |  |  |  |
| *Heyland 2015* |  |  |  |  |  |  |  |
| *Geense 2020* |  |  |  |  |  |  |  |
| *De Geer 2020* |  |  |  |  |  |  |  |
| *Papageorgiou 2018* |  |  |  |  |  |  |  |
| *Hope 2019* |  |  |  |  |  |  |  |
| *Montgomery 2019* |  |  |  |  |  |  |  |

**Additional file 1: Table S5:** Quality rating for the risk of bias using QUIPS

| **aHR^1^ (95% CI) for being frail (CFS 5-8)** | | | |
| --- | --- | --- | --- |
|  | **SOFA** | **SAPS II** | **APACHE II** |
| Complete case analysis | | | |
| **All patients** | 1.26 (1.15-1.38); p<0.0001 | 1.27 (0.94-1.71); p=0.1159 | 1.03 (0.92-1.15); p=0.64625 |
| **≥ 65 years** | 1.34 (1.25-1.44); p<0.0001 | 1.27 (0.94-1.71); p=0.1159 | 1.15 (1.06-1.25); p=0.00125 |
| Multiple imputation analysis | | | |
| **All patients** | 1.28 (1.15-1.41); p<0.0001 | 1.18 (1.05-1.34); p=0.00770 | 1.11 (0.96-1.29); p=0.17106 |
| **≥ 65 years** | 1.35 (1.26-1.45); p<0.0001 | 1.25 (1.15-1.36); p<0.0001 | 1.19 (1.09-1.3); p<0.0001 |
| aHR: Adjusted Hazard Ratio; ^1^ Reference CFS 1-3 ("fit") | | | |

**Additional file 1: Table S6:** aHR (95% CI) for being frail (CFS 5-8)

| **aHR^1^ (95% CI) for being vulnerable (CFS 4)** | | | |
| --- | --- | --- | --- |
|  | **SOFA** | **SAPS II** | **APACHE II** |
| Complete case analysis | | | |
| **All patients** | 1.07 (1.03-1.1); p=0.00054 | 1.08 (1-1.17); p=0.05698 | 1.02 (0.96-1.09); p=0.49418 |
| **≥ 65 years** | 1.1 (1.05-1.16); p=0.00029 | 1.08 (1-1.17); p=0.05698 | 1.07 (0.98-1.17); p=0.15195 |
| Multiple imputation analysis | | | |
| **All patients** | 1.07 (1.03-1.11); p=0.00139 | 1.03 (0.98-1.09); p=0.24218 | 1.01 (0.95-1.07); p=0.84884 |
| **≥ 65 years** | 1.1 (1.04-1.17); p=0.0006 | 1.06 (1-1.13); p=0.0620 | 1.04 (0.98-1.11); p=0.20012 |
| aHR: Adjusted Hazard Ratio; ^1^ Reference CFS 1-3 ("fit") | | | |

**Additional file 1: Table S7:** aHR (95% CI) for being vulnerable (CFS 4)

| All patients | | | | |
| --- | --- | --- | --- | --- |
| Complete Case Analysis – Model including SAPS II as severity index (n=2,256) | | | | |
|  |  | **HR^1^ (95%CI)** | | **P-value** |
| Frailty | vulnerable (CFS 4) | 1.08 (1-1.17) | | 0.05698 |
|  | frail (CFS 5-8) | 1.27 (0.94-1.71) | | 0.1159 |
| Severity | SAPS II (one point increase) | 1.03 (1.03-1.04) | | <0.0001 |
| Gender | male vs female | 0.92 (0.78-1.1) | | 0.35696 |
| Age | Age (5 years increase) | 1.35 (1.29-1.41) | | <0.0001 |
|  | | | | |
|  | | | | |
| Multiple Imputation Analysis - Model including SAPS II as severity index | | | | |
|  |  | **HR^1^ (95%CI)** | | **P-value** |
| Frailty | vulnerable (CFS 4) | 1.03 (0.98-1.09) | | 0.24218 |
|  | frail (CFS 5-8) | 1.18 (1.05-1.34) | | 0.00770 |
| Severity | SAPS II (one point increase) | 1.04 (1.03-1.05) | | <0.0001 |
| Gender | male vs female | 0.96 (0.9-1.01) | | 0.14310 |
| Age | Age (5 years increase) | 1.06 (1.02-1.1) | | 0.00724 |
|  | | | | |
|  | | | | |
| Complete Case Analysis – Model including APACHE II as severity index (n=14,084) | | | | |
|  | |  | |  |
|  |  | **HR^1^ (95%CI)** | | **P-value** |
| Frailty | vulnerable (CFS 4) | 1.02 (0.96-1.09) | | 0.49418 |
|  | frail (CFS 5-8) | 1.03 (0.92-1.15) | | 0.64625 |
| Severity | Apache II (one point increase) | 1.1 (1.07-1.12) | | <0.0001 |
| Gender | male vs female | 0.99 (0.93-1.06) | | 0.85712 |
| Age | Age (5 years increase) | 1.04 (1-1.08) | | 0.04515 |
|  | | | | |
|  | | | | |
| Multiple Imputation Analysis – Model including APACHE II as severity index | | | | |
|  |  | **HR^1^ (95%CI)** | | **P-value** |
| Frailty | vulnerable (CFS 4) | 1.01 (0.95-1.07) | | 0.84884 |
|  | frail (CFS 5-8) | 1.11 (0.96-1.29) | | 0.17106 |
| Severity | Apache II (one point increase) | 1.08 (1.05-1.11) | | <0.0001 |
| Gender | male vs female | 0.97 (0.91-1.04) | | 0.37384 |
| Age | Age (5 years increase) | 1.07 (1.03-1.11) | | 0.00026 |
|  | | | | |
|  | | | | |
| Patients ≥ 65 years | | | | |
| Complete Case Analysis – Model including SAPS II as severity index (n=2,256) | | | | |
|  |  | **HR^1^ (95%CI)** | **P-value** | |
| Frailty | vulnerable (CFS 4) | 1.08 (1-1.17) | 0.05698 | |
|  | frail (CFS 5-8) | 1.27 (0.94-1.71) | 0.1159 | |
| Severity | SAPS II (one point increase) | 1.03 (1.03-1.04) | <0.0001 | |
| Gender | male vs female | 0.92 (0.78-1.1) | 0.35696 | |
| Age | Age (5 years increase) | 1.35 (1.29-1.41) | <0.0001 | |
|  | | | | |
| Multiple Imputation Analysis – Model including SAPS II as severity index | | | | |
|  |  | **HR^1^ (95%CI)** | **P-value** | |
| Frailty | vulnerable (CFS 4) | 1.06 (1-1.13) | 0.0620 | |
|  | frail (CFS 5-8) | 1.25 (1.15-1.36) | <0.0001 | |
| Severity | SAPS II (one point increase) | 1.03 (1.03-1.04) | <0.0001 | |
| Gender | male vs female | 0.95 (0.88-1.02) | 0.14370 | |
| Age | Age (5 years increase) | 1.14 (1.09-1.19) | <0.0001 | |
|  | | | | |
| Complete Case Analysis – Model including APACHE II as severity index (n=6,264) | | | | |
|  |  | **HR^1^ (95%CI)** | **P-value** | |
| Frailty | vulnerable (CFS 4) | 1.07 (0.98-1.17) | 0.15195 | |
|  | frail (CFS 5-8) | 1.15 (1.06-1.25) | 0.00125 | |
| Severity | Apache II (one point increase) | 1.08 (1.05-1.11) | <0.0001 | |
| Gender | male vs female | 0.96 (0.92-1.01) | 0.08518 | |
| Age | Age (5 years increase) | 1.16 (1.08-1.26) | 0,00015 | |
|  | | | | |
| Multiple Imputation Analysis – Model including APACHE II as severity index | | | | |
|  |  | **HR^1^ (95%CI)** | **P-value** | |
| Frailty | vulnerable (CFS 4) | 1.04 (0.98-1.11) | 0.20012 | |
|  | frail (CFS 5-8) | 1.19 (1.09-1.3) | <0.0001 | |
| Severity | Apache II (one point increase) | 1.07 (1.05-1.09) | <0.0001 | |
| Gender | male vs female | 0.96 (0.9-1.03) | 0.23839 | |
| Age | Age (5 years increase) | 1.19 (1.15-1.24) | <0.0001 | |
|  | | | | |
| ^1^ Reference: "fit" (CFS 1-3) | | | | |

**Additional file 1: Table S8:** Regression analyses for ICU mortality, adjusted to APACHE II or SAPS II

| **SOFA (52)**  Sequential Organ Failure Assessment Score | **SAPS II (53)**  Simplified Acute Physiology Score II | **APACHE II (54)**  Acute Physiology and Chronic Health Evaluation II |
| --- | --- | --- |
| - Glasgow coma scale - Mean arterial pressure OR administration of vasopressors required - PaO2/FiO2 [mmHg (kPa)] - Platelets×103/μl - Bilirubin (mg/dl) [μmol/L] - Creatinine (mg/dl) [μmol/L] (or urine output) | - Age - Heart Rate - Systolic Blood Pressure - Temperature - Glasgow Coma Scale - Mechanical Ventilation or CPAP - PaO2 - FiO2 - Urine Output - Blood Urea Nitrogen - Sodium - Potassium - Bicarbonate - Bilirubin - White Blood Cell - Chronic diseases - Type of admission | **A**. Acute Physiology Score (measured within 24 hours of admission)   - AaDO2 or PaO2 (for FiO2≥0.5 or <0.5, respectively) - body temperature (rectal) - mean arterial pressure - blood pH - heart rate - respiratory rate - serum sodium - serum potassium - creatinine (Double point score for acute renal failure) - hematocrit - white blood cell count - Glasgow Coma Scale (15 minus actual GCS)   **B**. Age points  AGE (years) 🡺 Points   - ≤44 🡺 0 - 45-54 🡺2 - 55-64 🡺3 - 65-74 🡺5 - ≥75 🡺 6   **C.** Chronic health points  If the patient has a history of severe organ system insufficiency (i.e. liver cirrhosis, portal hypertension, NYHA class IV heart failure, severe respiratory disease, dialysis dependent) or is immunocompromised (i.e. due to chemotherapy, radiation, high dose steroid therapy, or advanced leukemia, lymphoma or AIDS) assign points as follows:   1. for nonoperative or emergency postoperative patients: 5 points 2. b. for elective postoperative patients: 2 points |

**Additional file 1: Table S9:** Overview on the different ICU-scores SOFA; SAPS II and APACHE II

| **Origin countries of the included data sets** |
| --- |
| Austria |
| Australia |
| Belgium |
| Canada |
| Croatia |
| Cyprus |
| Czech Republic |
| Denmark |
| France |
| Germany |
| Greece |
| India |
| Ireland |
| Italy |
| Libya |
| Norway |
| Poland |
| Portugal |
| Romania |
| Russia |
| Spain |
| Spain |
| Sweden |
| Switzerland |
| The Netherlands |
| Turkey |
| Ukraine |
| United Kingdom |
| USA |
| Wales |

**Additional file 1: Table S10:** Origin countries of the included data sets
